# NACSD RECOMMENDATIONS FOR OLDER ADULT DISASTER PREPAREDNESS, RESPONSE, AND RECOVERY

**DOI:** 10.1093/geroni/igad104.0687

**Published:** 2023-12-21

**Authors:** Lisa Brown, Anna Fisher, David Dosa, Sue Anne Bell

**Affiliations:** Palo Alto University, Palo Alto, California, United States; Hillcrest Health Services, Bellevue, Nebraska, United States; Brown University, Providence, Rhode Island, United States; University of Michigan, Ann Arbor, Michigan, United States

## Abstract

This presentation shares the recommendations drafted by the National Advisory Committee on Seniors and Disasters (NACSD). This federal advisory committee provides advice and guidance to the Assistant Secretary for Preparedness and Response within the U.S. Department of Health and Human Services (HHS) and the HHS Secretary. The NACSD is comprised of seven non-federal members with expertise in geriatric disaster planning, preparedness, response, or recovery and ten federal (non-voting) members who are ex officio representatives from agencies within HHS and other Executive Branch departments. The voting members drafted recommendations intended to address the immediate and persistent challenges in public health emergency preparedness, response, and recovery affecting older adults in the United States. Numerous meetings with federal and non-federal subject matter experts from various disciplines were held to develop the recommendations. The NACSD was also tasked with evaluating issues and programs and providing findings, advice, and recommendations to the Secretary of Health and Human Services to support and enhance all-hazards public health and medical preparedness, response, and recovery activities related to meeting the unique needs of older adults and their families across the entire spectrum of their wellbeing. The NACSD evaluated and provided input concerning the medical and public health needs of older adults related to preparation for, response to, and recovery from all-hazards emergencies; and may provide advice and consultation with respect to State emergency preparedness and response activities relating to older adults.

